# The mitochondrial phylogeny of an ancient lineage of ray-finned fishes (Polypteridae) with implications for the evolution of body elongation, pelvic fin loss, and craniofacial morphology in Osteichthyes

**DOI:** 10.1186/1471-2148-10-21

**Published:** 2010-01-25

**Authors:** Dai Suzuki, Matthew C Brandley, Masayoshi Tokita

**Affiliations:** 1 Department of Zoology, Graduate School of Science, Kyoto University, Sakyo, Kyoto, 606-8502 Japan; 2 Department of Ecology and Evolutionary Biology, Yale University, New Haven, CT 06520-8105 USA; 3 Graduate School of Life and Environmental Sciences, University of Tsukuba, Tsukuba, Ibaraki, 305-8572 Japan

## Abstract

**Background:**

The family Polypteridae, commonly known as "bichirs", is a lineage that diverged early in the evolutionary history of Actinopterygii (ray-finned fish), but has been the subject of far less evolutionary study than other members of that clade. Uncovering patterns of morphological change within Polypteridae provides an important opportunity to evaluate if the mechanisms underlying morphological evolution are shared among actinoptyerygians, and in fact, perhaps the entire osteichthyan (bony fish and tetrapods) tree of life. However, the greatest impediment to elucidating these patterns is the lack of a well-resolved, highly-supported phylogenetic tree of Polypteridae. In fact, the interrelationships of polypterid species have never been subject to molecular phylogenetic analysis. Here, we infer the first molecular phylogeny of bichirs, including all 12 recognized species and multiple subspecies using Bayesian analyses of 16S and cyt-b mtDNA. We use this mitochondrial phylogeny, ancestral state reconstruction, and geometric morphometrics to test whether patterns of morphological evolution, including the evolution of body elongation, pelvic fin reduction, and craniofacial morphology, are shared throughout the osteichthyan tree of life.

**Results:**

Our molecular phylogeny reveals 1) a basal divergence between *Erpetoichthys *and *Polypterus*, 2) polyphyly of *P. endlicheri *and *P. palmas*, and thus 3) the current taxonomy of Polypteridae masks its underlying genetic diversity. Ancestral state reconstructions suggest that pelvic fins were lost independently in *Erpetoichthys*, and unambiguously estimate multiple independent derivations of body elongation and shortening. Our mitochondrial phylogeny suggested species that have lower jaw protrusion and up-righted orbit are closely related to each other, indicating a single transformation of craniofacial morphology.

**Conclusion:**

The mitochondrial phylogeny of polypterid fish provides a strongly-supported phylogenetic framework for future comparative evolutionary, physiological, ecological, and genetic analyses. Indeed, ancestral reconstruction and geometric morphometric analyses revealed that the patterns of morphological evolution in Polypteridae are similar to those seen in other osteichthyans, thus implying the underlying genetic and developmental mechanisms responsible for those patterns were established early in the evolutionary history of Osteichthyes. We propose developmental and genetic mechanisms to be tested under the light of this new phylogenetic framework.

## Background

**Figure 1:**
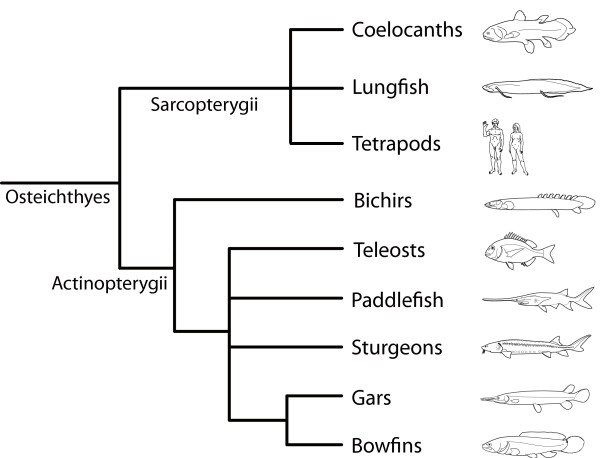
**Consensus view of the phylogeny of extant Osteichthyes, including the position of bichirs (Polypteridae), inferred from phylogenetic analyses of molecular and morphological data**.

Osteichthyans (bony fish and tetrapods; Fig. 1) have evolved remarkably diverse body plans since their initial radiation in the Late Silurian ~420 Mya [1,2]. It is therefore not surprising that most major extant lineages have been the subject of extensive evolutionary biology research. As a result, we know much about the evolutionary history and patterns of morphological evolution in osteichthyans, most notably teleost fish and tetrapods (amphibians, reptiles, and mammals). In the age of genomics and advanced molecular techniques, knowledge of these relationships and patterns has proven useful in uncovering the developmental and genetic mechanisms responsible for morphological diversity [e.g., [3-9]].

However, the same cannot be said for one lineage that diverged early in the evolutionary history of Actinopterygii (ray-finned fishes) - the family Polypteridae. Commonly known as "bichirs", this clade includes 12 extant described species (as well as multiple subspecies) that inhabit freshwater rivers and lakes of tropical Africa [10]. Little is known about polypterid evolution primarily because of their long history of phylogenetic and taxonomic confusion, likely owing to their unique collection of "primitive" (e.g., ganoid scales, cartilaginous skeleton, the intestine with a spiral valve) and derived (e.g., highly modified dorsal fins, pectoral fins with lobed base covered with scales, possession of only four gill arches) anatomical features [11-13]. However, recent morphological and molecular analyses have determined that bichirs are a basal lineage of Actinopterygii (ray-finned fishes; Fig. 1) [14-19].

Bichirs provide an attractive system to test whether patterns of morphological evolution are shared throughout the osteichthyan tree of life. If common patterns exist, it would suggest that the underlying genetic and developmental mechanisms responsible for those patterns were established early in the evolutionary history of Osteichthyes. For example, there exist multiple independent derivations of body elongation in modern Actinopterygian fish [e.g., [20-23]], amphibians [e.g., [24]], squamate reptiles [e.g., [25-27]], and numerous extinct tetrapod lineages [e.g., [28,29]]. The loss of pelvic fins and limbs has occurred multiple times over the course of osteichthyan evolution [22,27,30-32]. Furthermore, diverse changes in craniofacial morphology have been the subject of particularly extensive research [5,33-41].

However, the greatest impediment to elucidating the patterns of morphological change in Polypteridae is the lack of a well-resolved, highly-supported phylogenetic tree. In fact, the interrelationships of polypterid species have never been subject to molecular phylogenetic analysis. Those molecular studies that have included polypterid representatives did so in the context of determining "deep" osteichthyan and actinopterygian relationships (Fig. 1) or surveying *Hox *gene clusters [42].

Extant polypterids comprise two extant genera, *Erpetoichthys *and *Polypterus*. Boulenger [43] distinguished these two genera based on the extremely elongate body and absent pelvic fins in *Erpetoichthys*, and split *Polypterus *into two taxonomic groups based on the position of the mandible relative to the snout ("lower jaw protrusion" hereafter). Poll [44-46] defined five clusters of species and an ancestral species within extant polypterids based on anatomical features such as relative jaw length, the location and size of the eyes, width of the suboperculum, and proportion of the gular plate. An analysis of 15 anatomical characters suggested, among other things, a sister relationship between *Erpetoichthys *and *P. weeksi*, thus rendering *Polypterus *polyphyletic [47]. However, this study did not incorporate objective rooting or optimality criteria and subsequent parsimony reanalysis revealed that this phylogeny is largely unresolved [12]. These conflicting and inconclusive results warrant additional phylogenetic scrutiny using molecular data.

Here, we infer the first molecular phylogeny of Polypteridae using all 12 recognized species including all but three recognized subspecies to achieve three major objectives. First, we evaluate whether the current species taxonomy underestimates the underlying phylogenetic diversity. Secondly, we examine patterns of evolution in body elongation pelvic fin reduction, and craniofacial morphological evolution in this new phylogenetic context. Finally, we recommend developmental and genetic mechanisms to be tested in the future in conjuction with this new phylogenetic framework.

## Methods

### Phylogenetic analyses

We obtained fresh tissues from commercial dealers of 15 recognized species and subspecies of polypterids including *Erpetoichthys calabaricus*, *Polypterus ansorgii*, *P*. *bichir bichir*, *P. b. lapradei*, *P. delhezi*, *P. endlicheri congicus*, *P. e. endlicheri*, *P. mokelembembe*, *P. ornatipinnis*. *P. palmas buettikoferi*, *P. p. polli*, *P. retropinnis*, *P. senegalus senegalus*, *P. teugelsi *and *P. weeksii *(Table 1). One to four specimens of each taxon were included in the phylogenetic analyses. Although we included all recognized species of polypterids, we were unable to obtain tissue samples for three subspecies, *P. bichir katangae*, *P. palmas palmas*, and *P. senegalus meridionalis *(Table 1). We obtained DNA sequences of outgroup taxa (Sturgeon [*Acipenser transmontanus*], Bowfin [*Amia calva*], Coelacanth [*Latimeria chalumnae*], Spotted gar [*Lepisosteus oculatus*], Paddlefish [*Polyodon spatula*], and Lungfish [*Protopterus dolloi*]) from GenBank (Additional file 1).

**Table 1:** Number of vertebrae and condition of the lower jaw and pelvic fins in each species and subspecies of Polypteridae.

Species	N. of vertebrae	Jaw protrusion	Pelvic fin
*Erpetoichthys calabaricus*	110-113 (111.5)	Upper	Absent
*Polypterus ansorgii*	57	Lower	Present
*P. bichir bichir*	67	Lower	Present
P. bichir katangae*	?	Lower	Present
*P. bichir lapradei*	60-62 (61)	Lower	Present
*P. delhezi*	55	Upper	Present
*P. endlicheri congicus*	57	Lower	Present
*P. endlicheri endlicheri*	53-57 (55)	Lower	Present
*P. mokelembembe*	56	Upper	Present
*P. ornatipinnis*	56-60 (58)	Upper	Present
P. palmas palmas*	56-59	Upper	Present
*P. palmas buettikoferi*	56-58 (57)	Upper	Present
*P. palmas polli*	50-56 (53)	Upper	Present
*P. retropinnis*	57-58 (57.5)	Upper	Present
P. senegalus meridionalis*	?	Upper	Present
*P. senegalus senegalus*	53-59 (56)	Upper	Present
*P. teugelsi*	63-65 (64)	Upper	Present
*P. weeksii*	57	Upper	Present

Values in parentheses are means of vertebral number used in the squared-change parsimony ancestral state reconstruction analyses. *Taxa not sampled in this study.

DNA samples were extracted following the same procedure used in Tokita et al. [48]. A part of the mitochondrial 16S rRNA and cytochrome b (cyt b) genes were amplified by PCR System GeneAmp 2700 (Applied Biosystems, Lincoln, USA) using an Ex *Taq *polymerase kit (Takara Shuzo Co., Ltd., Otsu, Japan) and the primers provided in Additional file 2. After initial denaturing for 5 min at 95°C, 25-30 cycles were performed with denaturing 1 min at 94°C, annealing 3 min at 50-60°C, and primer extension for 3 min at 72°C, followed by a final elongation of 7 min at 72°C. PCR products were purified by PEG/NaCl precipitation and then sequenced using a Big Dye Terminator Cycle Sequencing Ready Reactions Kit v1.0 and an ABI PRISM 377 and 3130 DNA Sequencer (Applied Biosystems, Lincoln, USA), using the primers provided in Additional file 2.

Because of conserved codon reading frames, the cyt b protein coding sequences could be unambiguously aligned by eye. The 16S rRNA was also aligned by eye, but nucleotide positions that could not be unambiguously aligned were excluded from phylogenetic analysis (data set available from the authors). In some regions of the 16S data, the ability to align the polypterid taxa was improved if we removed sequences for the outgroup taxa (and their corresponding data replaced with "?" in these regions). Because our primary goal is to test the interrelationships of polypterids, and not necessarily their relationship to other osteichthyans, we feel the exclusion of these data is justified. All DNA sequences were deposited in GenBank (Additional file 1). The final data set for subsequent phylogenetic analysis included 925 base pairs (bp) of 16S (256 parsimony informative characters) and 932 bp of cyt b (103, 29, and 292 parsimony informative characters for the first, second, and third codon positions), for a total of 1857 total characters.

We employed partitioned Bayesian analyses to infer the phylogenetic relationships among polypterids. Previous studies have demonstrated that applying separate models of nucleotide evolution to specific subsets of nucleotide data (i.e., "partitioned" or "mixed-model" analyses) improves phylogenetic reconstruction [49-51]. We therefore partitioned the data *a priori *into four total partitions, one for the 16S rRNA and three for each codon position of cyt b. We determined the appropriate models of sequence evolution from a pool of 16 models (K80, HKY, SYM, GTR with and without I and Γ) for each partition using a JC corrected neighbor-joining tree in PAUP* [52] and the Bayesian Information Criterion [BIC: [53]]. We subsequently conducted Bayesian phylogenetic analyses using the most appropriate models (GTR+I+Γ, GTR+I+Γ, HKY+I+Γ, and GTR+Γ for the 16S and three cyt b codon positions, respectively) in the parallel version of MrBayes v3.1.1 [54,55]. Each Bayesian analysis consisted of 10^7 ^generations, using four chains sampled every 1000 generations and default priors (substitution rates, Dirichlet [1, 1, 1, 1, 1, 1]; base frequencies, Dirichlet [1, 1, 1, 1]; gamma shape parameter, uniform [0, 200]; topologies, uniform; branch lengths, unconstrained exponential [λ = 10]).

To determine convergence of the Bayesian analyses, we constructed cumulative posterior probability plots for each clade using the *cumulative *function in AWTY [56]. Stationarity was assumed when the cumulative posterior probabilities of all clades stabilized. These plots indicated that excluding the first two million generations as burn-in was sufficient. To decrease the chance of reaching apparent stationarity on local optima, four separate analyses were performed. Posterior probability estimates for each clade were then compared between the four analyses using a scatter-plot created by the *compare *command in AWTY. Posterior probability estimates for clades were similar in all four analyses, and the results of the analyses were combined and the frequency of inferred relationships in these 32,000 trees represented estimated posterior probabilities of clades. Posterior probabilities ≥ 0.95 are considered statistically significant (i.e., "strong") clade support [57].

### Morphometric analysis

To investigate shape differences in the head region among *Polypterus *species, we employed landmark-based geometric morphometrics [58,59]. This approach allows us to describe morphological transformation of certain organismic structures visually and compare morphological differences among taxa quantitatively [59] and has been widely used in contemporary evolutionary biology research [e.g., [34,40,41]]. A total of 13 taxa and 26 specimens caught in wild were included in this analysis. Photographs of both dorsal and lateral views were taken with a digital camera. The photo images of the specimens were imported into the tpsDig2 software [60]. Eight and ten landmarks were placed for dorsal and lateral views, respectively (Additional file 3). The generalized least squares Procrustes superimposition, which removes information unrelated to shape such as position, scale, and orientation, was carried out using Coordgen 6n [61]. Based on the coordinates after the Procrustes superimposition, the partial warp scores were computed and principal component analysis (PCA) was conducted by calculating PCs based on the covariance matrix derived from the partial warp scores using PCAgen6 h [62].

### Ancestral state reconstruction

To evaluate the evolution of body elongation and pelvic fin loss within polypterids, we reconstructed ancestral states using data from extant sampled taxa, the rooted mtDNA phylogram (using mean branch lengths from the posterior distribution) estimated from our Bayesian phylogenetic analyses (above), and Mesquite v2.6 [63]. We removed the non-polypterid outgroups and pruned the data set and trees to include a single exemplar of each species and subspecies. We collected vertebral number for each species from FishBase [64] and preserved specimens. Polypterid species typically exhibit variation in the number of vertebrae (Table 1). Because there is currently no statistically elegant way to incorporate this information into ancestral state reconstruction analyses (e.g., modeling vertebral number in each species as statistical distributions) we used the mean number of vertebrae and coded it as a continuous character [e.g., [65]]. Ancestral vertebrae number for each node was subsequently estimated using weighted squared-change parsimony [66]. Presence of pelvic fin was coded as discrete binary characters and the marginal probabilities of each state at each node were estimated with maximum likelihood [67,68] using the 1-parameter symmetrical Markov model [69]. Ancestral states with a marginal probability ≥ 0.95 were considered resolved with statistical significance.

## Results and Discussion

### Polypterid phylogeny and hidden diversity

**Figure 2:**
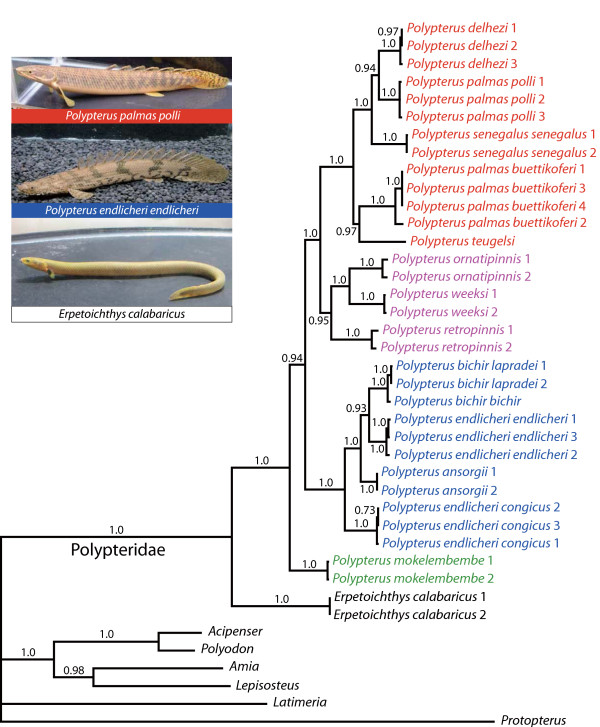
**Molecular phylogeny of the extant polypterid species inferred from partitioned Bayesian analyses 16SrRNA and cyt b mitochondrial genes**. Branch lengths are means of the posterior distribution. Numbers above or below the node indicate the Bayesian posterior probability that clade is correctly estimated given the model. Posterior probabilities less than 0.50 are not shown. Colors indicate groups defined in Fig. 4.

Posterior probabilities (PP) for a vast majority of nodes are statistically significant (≥ 0.95) and the clades with probabilities less than 0.90 are relationships between populations of subspecies (Fig. 2). The analyses support a basal split between *Erpetoichthys *and *Polypterus*. In *Polypterus*, the recently described *P. mokelembembe *[69] is the sister taxon to all other members of the genus; although support for this relationship is very high (PP = 0.94), it is not significant. The remaining *Polypterus *are divided into two major clades, one of which (*P. bichir + P. endlicheri + P ansorgii*) is characterized by lower jaw protrusion (Table 1; see below).

These results reinforce the findings of Boulenger [43] who recognized *Erpetoichthys *and *Polypterus *as distinct genera, in addition to two groups within *Polypterus *based on the condition of the lower jaw (*P. mokelembembe *was not yet described). The phylogenetic position of *Erpetoichthys *is not particularly surprising considering numerous biological aspects of this species. The skull of *E. calabaricus *possesses a variety of unique characteristics that are not found in *Polypterus *[12]. Furthermore, the ecology of *E. calabaricus *quite different than *Polypterus *as it inhabits coastal and estuarine areas unlike *Polypterus *species that primarily inhabit freshwater [10,70-72].

Our data support Boulenger's conclusion of a close relationship between *P. weeksi *and *P. ornatipinnis *[43]. However, the present analyses reveal phylogenetic relationships that are substantially different from other previous studies focusing on the detailed interrelationships of the species. Analyses of 15 morphological and anatomical traits [47] identified close relationships among *P. ornatipinnis*, *P. weeksii*, and *Erpetoichthys calabaricus*, and among *P. senagalus*, *P. retropinnis*, *P. palmas *(although subsequent parsimony reanalysis of these data inferred an almost completely unresolved tree [12]). However, neither of these relationships was inferred by our analyses.

Poll [44-46] recognized close relationships among *P. ornatipinnis*, *P. weeksii*, and *P. delhezi *and that between *P. senagalus *and *P. retropinnis *based on morphological characteristics (e.g., relative jaw length, the location and size of the eyes). Our results statistically reject these phylogenetic hypotheses (i.e., alternative hypotheses have a posterior probability > 0.95). Our phylogenetic analyses also statistically reject Poll's hypothesis [45] that *P. ansorgii *is the sister lineage to all other *Polypterus *instead, this position is represented by *P*. *mokelembembe*.

Our phylogenetic analyses also demonstrate that at least two species, as currently described, are not natural groups - *P. endlicheri *and *P. palmas*. *Polypterus endlicheri *is paraphyletic with respect to both *P. bichir *and *P. ansorgii*. Taxonomic changes are clearly needed and we recognize *P. e. congicus *and *P. e. endlicheri *as distinct species, *P. congicus *and *P. endlicheri*. Hanssens et al. [73] recognized three subspecies in *P. palmas*: *P. p. palmas*, *P. p. polli*, *P. p. buettikoferi *based on morphometrical inferences. In our phylogeny, the sister relationship between *P. p. polli *and *P. delhezi *is strongly supported (PP = 1.0) as well as the relationship between *P. p. buettikoferi *and *P. teugelsi *(PP = 1.0). Thus, the morphometric resemblance between *P. p. buettikoferi *and *P. p. polli *is clearly the outcome of evolutionary convergence. To understand the evolutionary history of *P. palmas *complex and make subsequent taxonomic changes, it is necessary to include *P. p. palmas *in future phylogenetic analyses.

These results imply that morphological characteristics such as body proportion and body coloration used in previous studies are not suitable for inferring phylogeny, possibly because states of these characters tend to be flexibly altered depending on the condition of the species' environment. Furthermore, these phylogenetic results not only have implications for morphological evolution within Polypteridae (below), but also reveal the hidden genetic diversity of this ancient clade of enigmatic fish. That at least two species are polyphyletic (*P. endlicheri *and *P. palmas*) is evidence that the current taxonomy of *Polypterus *underestimates its genetic diversity.

Perhaps more importantly, we find that *P. mokelembembe*, a species only recently described [74], represents the sister lineage to all other members of the genus. This result suggests that much of the evolutionary diversity of extant Polypteridae has yet to be fully discovered.

As both the 16S and cyt b mitochondrial genes are maternally inherited as a single unit, we acknowledge that our phylogeny specifically tracks the evolutionary history of the mitochondrial genome, and not necessarily the species history. However, we note that there are no obvious instances of recent introgression (e.g., [75]) as all of the named taxa are monophyletic. However, we cannot exclude the possibility that incomplete lineage sorting obscures deeper relationships (see [76] for a review), but we also note that this is unlikely given the rapid coalescence of mitochondrial DNA. Furthermore, we note that, although we have not sampled the entire mitochondrial genome for this analysis, the fact that almost every node in our phylogeny is significantly supported (posterior probability > 0.95) is evidence that we have indeed captured almost as much "phylogenetic signal" as is possible from the mitochondrial genome. Nonetheless, for clarity, we refer to the phylogeny as the "mitochondrial phylogeny" to distinguish it from the true (albeit unknowable) species tree, and acknowledge that a more confident species tree of polypterids awaits future analyses of many more independently evolving loci.

### Evolution of body elongation

**Figure 3:**
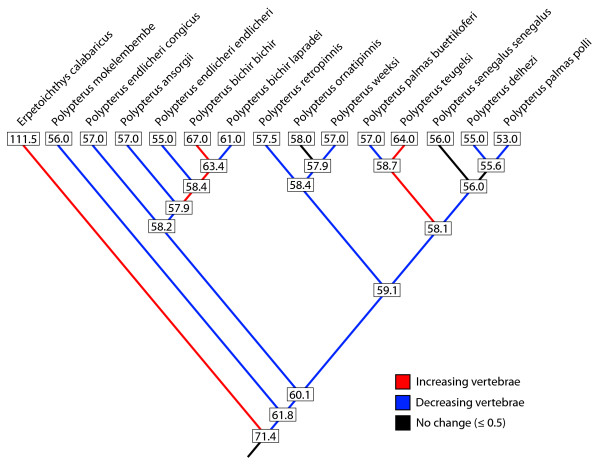
**Extant states and result of ancestral state reconstructions of vertebral number using squared-change parsimony**.

Our ancestral state reconstructions (Fig. 3) estimate numerous increases and decreases of vertebral number, and thus, changes in body elongation. However, the most striking transformation is seen in *Erpetoichthys*. *Erpetoichthys *possesses between 110 and 113 vertebrae (Table 1), but the vertebral number reconstruction for the most recent common ancestor (MRCA) of this genus and *Polypterus *is ~71. It is not known whether the already relatively high number of vertebrae reconstructed for the MRCA is an artifact of the extremely high number of vertebrae in *Erpetoichthys *biasing the estimation of the root, or evidence that extinct stem polypterids were already relatively elongate. Regardless, *Erpetoichthys *is much more elongate than the estimated common ancestor of crown polypterids.

Anguilliformity (eel-like elongated body form), such as that seen in *Erpetoichthys*, is seen across diverse lineages of vertebrates including ray-finned fishes, caecilian amphibians, and squamate reptiles [21,23-27,77], and an increase in vertebral number is considered as the common primary factor underlying such body form change [22]. Developmentally, vertebrae emerge from 'somites' that form one after another, with antero-posterior direction in the embryo. They bud off from the anterior end of unsegmented 'presomitic mesoderm' and this budding is regulated by a segmentation clock represented by oscillation of 'clock' genes such as *Lunatic fringe *[reviewed in [78,79]]. Recently, it was found that the tempo of the segmentation clock is accelerated in snake embryos, "ticking" around four times faster relative to growth rate than in shorter-bodied animals like chick and mouse and consequently generating a large number of somites [80].

*Erpetoichthys *has around 100 preanal vertebrae, which is almost double the number of other polypterid species [[22], pers. obs.]. Although few embryological studies of *Erpetoichthys *have been conducted [81,82], the yolk-sac larva and apterolarva of this species appear to be much more elongated and have more somites than those of *Polypterus*. It is plausible that an increase of vertebrate number in *Erpetoichthys *was brought about by a similar developmental mechanism to that exploited in snake embryogenesis [80,83], although the precise molecular mechanisms that regulate tempo of segmentation clock in vertebrate embryos have not been identified yet. The present study implies that body elongation observed in *Erpetoichthys *seems to be brought about in an early phase of polypterid evolution, possibly by accelerating the tempo of the segmentation clock twice as fast as that in other polypterids.

Although the extreme degree of body elongation observed in *Erpetoichthys *is not found in extant *Polypterus*, two species: *P. bichir *and *P. teugelsi *show a relative increase of vertebrate number (Table 1; Fig. 3). Although the total number of vertebrae is over 60 in both species, the mechanisms by which they achieve this body elongation are quite different. In *P. bichir*, the number of preanal vertebrae is less than 50 like that in majority of *Polypterus *species but the number of postanal vertebrae increases into over 14 [22]. On the other hand, *P. teugelsi *shows relative elongation of the trunk region, possessing at least 54 preanal vertebrae (pers. obs.). Instead, this species has only 10 postanal vertebrae keeping the condition in majority of *Polypterus *species. Thus, body elongation seems to have been achieved independently in two *Polypterus *species by employing different developmental mechanism that functions in separate developmental modules [22]. We note that this pattern is surprisingly similar to that seen in squamate reptiles (lizards and snakes) in which body elongation is also achieved by two distinct mechanisms elongating either the trunk or the tail [24-26].

### Pelvic fin loss

In vertebrates, the body elongation tends to co-occur with a loss or reduction of paired appendages [21,24-26,29,84,85]. Following this evolutionary trend, *Erpetoichthys*, whose body is extremely elongated, also lacks pelvic fins (Table 1). Maximum likelihood ancestral state reconstructions strongly support the conclusion that pelvic fins were present in the most recent common ancestor of *Erpetoichthys *and *Polypterus *(Marginal probability = 0.97; data not shown), and that they were secondarily lost in the *Erpetoichthys *lineage. Given that the loss of pelvic appendages is frequent in multiple osteichthyan lineages, it is likely that the genetic mechanism responsible for pelvic fin loss in *Erpetoichthys *is similar to other members of this group. Translocation of *Hox *gene expression in trunk paraxial and/or lateral plate mesoderm is known to be the potential mechanism underlying limb elimination in vertebrates [[86,87]; but see recent paper by Wolstering et al. [88]]. However, this is a less likely explanation for pelvic fin loss in *Erpetoichthys *because such alteration of developmental program might also obliterate any vestiges of the pelvic girdle yet *Erpetoichthys *retains a pair of pelvic girdle elements (pers. obs.). Degeneration of apical ectodermal ridge (AER) and lack of expression of AER-associated genes might account for hindlimb truncation in lizards [89,90] and snakes [86], but this has never been recorded in non-tetrapods. Similarly, failure in establishing the Zone of Polarizing Activity (ZPA) has only been implicated in hindlimb loss in cetaceans [91]. The most promising potential mechanism responsible for pelvic fin loss in *Erpetoichthys *is the elimination of expression of transcription factor-encoding genes such as *Pitx1 *and *Hoxd9 *in the pelvic region - the mechanism that causes pelvic fin loss in some teleost fishes [31,32,92]. Unfortunately, the pattern of pelvic fin reduction in embryogenesis of *Erpetoichthys *has not been described precisely [82] and thus, discrimination of these various hypotheses is not yet possible. However, future studies of these genetic and developmental mechanisms can do so within the phylogenetic framework constructed here.

### Transformation of craniofacial morphology

The first three PCs of the geometric morphometrical analysis of both dorsal and lateral views of the head accounted for over 70% of the total shape variation. In the comparison of the dorsal views, PC1 explains 36% of the total variance and primarily describes positional change of the orbit. PC2 explains 21% of the total variance (Additional file 4). In the comparison of the lateral views, PC1 explains 51% of the total variance and describes positional change of the tip of the mandible, the orbit, and labial end. While, PC2 explaining 14% of the total variance describes positional change of the opercle.

**Figure 4:**
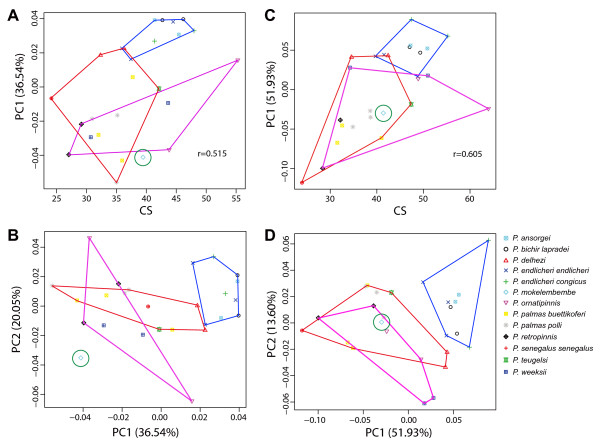
**Plots of principal component (PC) and centroid size (CS) for morphometric characters of *Polypterus***. a) CS and PC1 for dorsal view, b) PC1 and PC 2 for dorsal view, c) CS and PC1 for ventral view, d) PC1 and PC2 for ventral view. Grouping corresponds to the clade inferred from molecular phylogenetic analysis (Fig. 2): Blue = *Polypterus ansorgei*, *P. bichir lapradei*, *P. endlicheri endlicheri*, and *P. e. congicus*; Green = *P. mokelembembe*; Purple = *P. ornatipinnis*, *P. retropinnis*, and *P. weeksii*; Red = *P. delhezi*, *P. palmas buettikoferi*, *P. p. polli*, *P. senegalus senegalus*, and *P. teugelsi*.

Our morphometric analysis revealed the trend of morphological change in *Polypterus *species where PC1 positively correlates with the size of the head (Fig. 4a and 4c). Phylogenetically closely-related *P*. *ansorgii*, *P*. *bichir *and *P*. *endlicheri *occupied the most extreme positions on the positive sides of both PC1 and PC2 axes. This suggests that these three species not only tend to have a rostrally-projected lower jaw (a condition already noted by previous authors [43,47]), but also a medially located and up-righted orbit, enlarged mouth, and rostroventral distortion of the opercle. Judging from our mitochondrial phylogeny, the character complex seen in these three species is a derived condition.

Interestingly, *P*. *delhezi *displayed a similar tendency of morphological change in PC1 axis of both dorsal and lateral views, indicating that this species actually possesses an intermediate condition where neither the upper or lower jaw show pronounced rostral projection. Because *P*. *delhezi *is distantly related to the lower jaw projection clade (*P*. *ansorgii*, *P*. *bichir *and *P*. *endlicheri*), the similar cranial shape seen in two polypterid lineages has apparently evolved independently. In polypterid species whose snout projected more rostrally to the tip of mandible, the range of variation was broader than that in species with lower jaw protrusion (Fig. 4). Furthermore, the range was broadly overlapped between two main clades: the monophyletic group composed of *P*. *ornatipinnis*, *P*. *weeksii*, and *P*. *retropinnis *(purple) and that composed of *P*. *delhezi*, *P*. *palmas, P*. *senegalus*, and *P*. *teugelsi *(red), showing no lineage-specific pattern of craniofacial transformation.

There are multiple potentially productive avenues of future genetic and developmental research into the mechanisms of craniofacial development in Polypteridae. In vertebrates, morphology of the lower jaw is regulated by cranial neural crest cells, and several transcription factors in BMP and/or FGF signaling pathway appear to be involved in the process [4,38,93-97]. Although we do not know what change in developmental program caused substantial transformation of craniofacial morphology in *P*. *ansorgii*, *P*. *bichir *and *P*. *endlicheri*, the event that occurred in relatively early phase of polypterid evolution might have facilitate the shift into a new ecological niche, affecting their feeding strategy.

## Conclusion

The discovery of patterns of morphological evolution among "deep" lineages of organisms permits further exploration of the general underlying genetic and developmental mechanisms that evolved early in evolutionary history. Our new molecular phylogeny of Polypteridae, in conjunction with ancestral state reconstruction and geometric morphometric analyses reveals that many of the patterns of morphological evolution seen in polypterids are shared with other actinopterygian, and indeed, osteichthyan lineages. Thus, this implies the underlying genetic and developmental mechanisms responsible for those general patterns were established early in the evolutionary history of Osteichthyes, or perhaps even older in the craniate tree of life. Moreover, these patterns suggest interesting avenues of future evolution and developmental research that are only possible in a phylogenetic framework.

## Authors' contributions

MT designed the study. DS and MT collected data. DS and MT performed the morphometric analyses and MCB performed the phylogenetic analyses. MT wrote the paper with the assistance of DS and MCB. All authors read and approved the final manuscript.

## Supplementary Material

Additional file 1 Information of DNA sequences analyzed in this study. Click here for file

Additional file 2 List of primers used in this study. Click here for file

Additional file 3 **Landmark definition for morphometric analysis**. (A) *Polypterus palmas buettikoferi *in dorsal view, (B) *P. endlicheri congicus *in dorsal view, (C) *P. p. buettikoferi *in lateral view, (D) *P. e. congicus *in lateral view. Click here for file

Additional file 4 **Thin-plate splines (TPS) of principal component 1 (PC1) and 2 (PC2) for both dorsal and ventral views of the head of Polypterus**. Arrows indicate PC value plus positive 0.1 score. (A) PC1 in dorsal view, (B) PC2 in dorsal view, (C) PC1 in lateral view, (D) PC2 in lateral view. Numbers along with each plot indicate landmarks defined in Additional file 3. Click here for file
