# The mitochondrial phylogeny of an ancient lineage of ray-finned fishes (Polypteridae) with implications for the evolution of body elongation, pelvic fin loss, and craniofacial morphology in Osteichthyes

**DOI:** 10.1186/1471-2148-10-209

**Published:** 2010-07-12

**Authors:** Dai Suzuki, Matthew C Brandley, Masayoshi Tokita

**Affiliations:** 1 Department of Zoology, Graduate School of Science, Kyoto University, Sakyo, Kyoto, 606-8502 Japan; 2 Department of Ecology and Evolutionary Biology, Yale University, New Haven, CT 06520-8105 USA; 3 Graduate School of Life and Environmental Sciences, University of Tsukuba, Tsukuba, Ibaraki, 305-8572 Japan

## Correction

After re-evaluation, we have determined that two species, *Polypterus retropinnis *and *P. mokelembembe*, were misidentified in our original study [1]. The overall morphology of both species is very similar, to the point that re-examination of the type series of *P. retropinnis *demonstrated that it consisted of both *P. retropinnis *and *P. mokelembembe *[2]. Therefore, the placement of these two taxa in our published phylogeny should be switched (Fig. 1 below; Fig. two in the original study). This error has only minor impact on our analyses of pre-sacral vertebrate evolution (Fig. 2; Fig. three in the original study) and cranio-facial morphology (Fig. 3; Fig. four in the original study).

**Figure 1:**
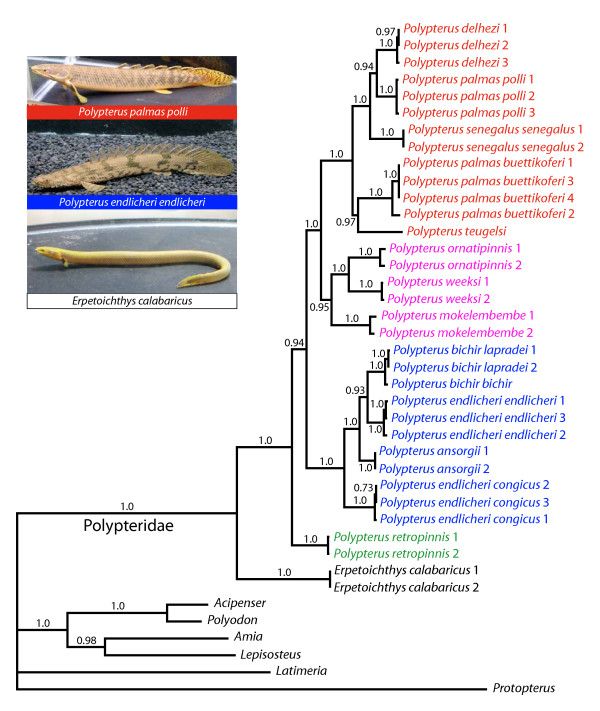
**Molecular phylogeny of the extant polypterid species inferred from partitioned Bayesian analyses 16SrRNA and cyt b mitochondrial genes**. Branch lengths are means of the posterior distribution. Numbers above or below the node indicate the Bayesian posterior probability that clade is correctly estimated given the model. Posterior probabilities less than 0.50 are not shown. Colors indicate groups defined in Fig. four. Please note that this corrected figure corresponds to Fig. two of the original study.

**Figure 2:**
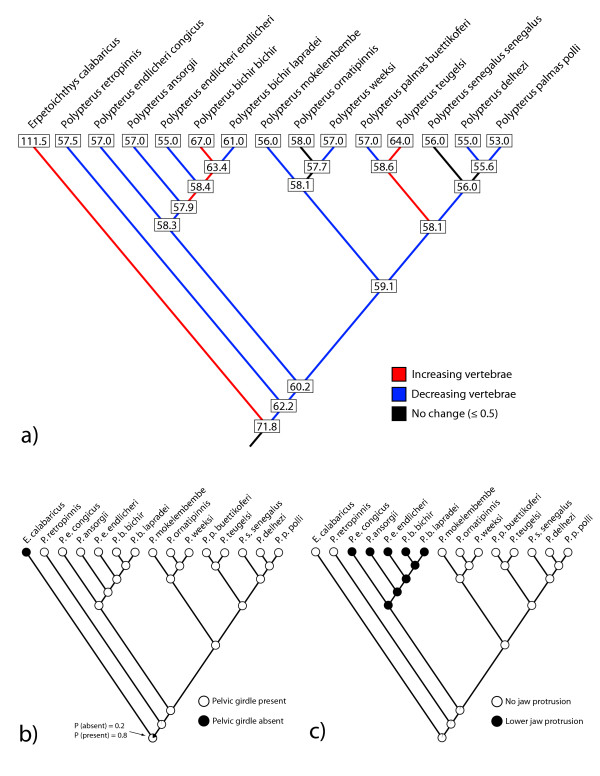
**Extant states and result of ancestral state reconstructions of vertebral number using squared-change parsimony**. Please note that this corrected figure corresponds to Fig. three of the original study.

**Figure 3:**
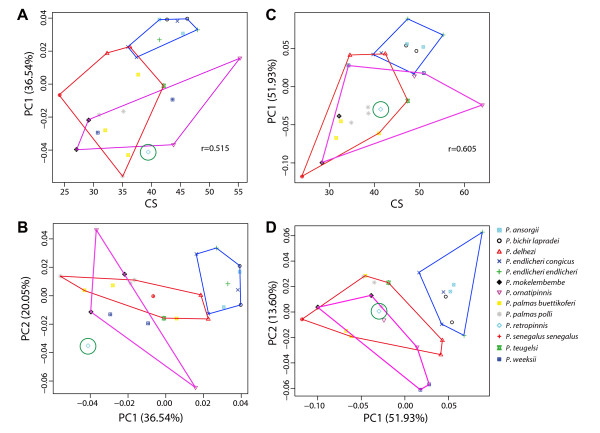
**Plots of principal component (PC) and centroid size (CS) for morphometric characters of *Polypterus***. a) CS and PC1 for dorsal view, b) PC1 and PC 2 for dorsal view, c) CS and PC1 for ventral view, d) PC1 and PC2 for ventral view. Grouping corresponds to the clade inferred from molecular phylogenetic analysis (Fig. two): Blue = *Polypterus ansorgei*, *P. bichir lapradei*, *P. endlicheri endlicheri*, and *P. e. congicus*; Green = *P. retropinnis*; Purple = *P. ornatipinnis*, *P. mokelembembe*, and *P. weeksii*; Red = *P. delhezi*, *P. palmas buettikoferi*, *P. p. polli*, *P. senegalus senegalus*, and *P. teugelsi*. Please note that this corrected figure corresponds to Fig. four of the original study.

We note that any reference in the original text to *P. retropinnis *is in fact referring to *P. mokelembembe*, and vice versa.

Secondly, in our published cranio-facial morphology figure (Fig. four in the original study), the symbols for *P. endlicheri congicus *and *P. e. endlicheri *were switched. We have corrected this below (Fig. 3). Because both taxa are characterized by lower jaw protrusion, this correction does not substantially change our conclusions.
