# Supplementary material for: The mitochondrial phylogeny of an ancient lineage of ray-finned fishes (Polypteridae) with implications for the evolution of body elongation, pelvic fin loss, and craniofacial morphology in Osteichthyes
Source: BMC Evol Biol. 2010 Jan 25;10:21. doi: 10.1186/1471-2148-10-21 (PMC2825197; doi:10.1186/1471-2148-10-21)
Supplement: Additional file 1 — Information of DNA sequences analyzed in this study. [file 1471-2148-10-21-S1.PDF]

Additional file 1

SPECIES	ACCESSION NUMBER	REFERENCES
Latimeria chalumnae	AB257296	Sasaki,T., Sato,T., Miura,S., Bwathondi,P.O.J., Ngatunga,B.P. and Okada,N. Mitogenomic analysis for coelacanths (Latimeria chalumnae) caught in Tanzania. Gene 389, 73–79 (2007)
Acipenser transmontanus	AB042837	Inoue,J.G., Miya,M., Tsukamoto,K. and Nishida,M. Basal actinopterygian relationships: a mitogenomic perspective on the phylogeny of the 'ancient fish'. Mol. Phylogenet. Evol. 26, 110–120 (2003)
Amia calva	AB042952	Inoue,J.G., Miya,M., Tsukamoto,K. and Nishida,M. Basal actinopterygian relationships: a mitogenomic perspective on the phylogeny of the 'ancient fish'. Mol. Phylogenet. Evol. 26, 110–120 (2003)
Lepisosteus oculatus	AB042861	Inoue,J.G., Miya,M., Tsukamoto,K. and Nishida,M. Basal actinopterygian relationships: a mitogenomic perspective on the phylogeny of the 'ancient fish'. Mol. Phylogenet. Evol. 26, 110–120 (2003)
Polyodon spatula	AP004353	Inoue,J.G., Miya,M., Tsukamoto,K. and Nishida,M. Basal actinopterygian relationships: a mitogenomic perspective on the phylogeny of the 'ancient fish'. Mol. Phylogenet. Evol. 26, 110–120 (2003)
Protopterus dolloi	L42813	Zardoya,R. and Meyer,A. The complete nucleotide sequence of the mitochondrial genome of the lungfish (Protopterus dolloi) supports its phylogenetic position as a close relative of land vertebrates. Genetics 142 (4), 1249–1263 (1996)
