# Supplementary material for: The mitochondrial phylogeny of an ancient lineage of ray-finned fishes (Polypteridae) with implications for the evolution of body elongation, pelvic fin loss, and craniofacial morphology in Osteichthyes
Source: BMC Evol Biol. 2010 Jan 25;10:21. doi: 10.1186/1471-2148-10-21 (PMC2825197; doi:10.1186/1471-2148-10-21)
Supplement: Additional file 2 — List of primers used in this study. [file 1471-2148-10-21-S2.PDF]

Additional file 2

PRIMER NAME	GENES	PRIMER SEQUENCES	REFERENCES
PolycytbF	mtDNA cytb	5'-ATGGCMATCAYMCGYAAACCCACCC-3'	this study
PolycytbR	mtDNA cytb	5'-GGRTCTTCTACKGGYTGTCCTCC-3'	this study
L2206s	mtDNA 16s	5'-GGCCTAAAAGCAGCCACCTGTAAAGACAG-3'	Honda, M., Ota, H., Kobayashi, M., Nabhitabhata, J., Yong, H.-S., Hikida, T., 1999. Phylogenetic relationships of the frying lizards genus Draco (Reptilia, Agamidae). Zool. Sci. 16, 535-549.
polypterus16sF1+	mtDNA 16s	5'-GCCACCTGTAAAGACAG-3'	this study
16SR.0	mtDNA 16s	5'-TAGATAGAAACCGACCTGGATT-3'	Whiting, A.S., Bauer, A.M., Sites Jr., J.W., 2003. Phylogenetic relationships and limb loss in sub-Saharan African scincine lizards (Squamata: Scincidae). Mol. Phylogenet. Evol. 29, 582-598.
