# Supplementary material for: The mitochondrial phylogeny of an ancient lineage of ray-finned fishes (Polypteridae) with implications for the evolution of body elongation, pelvic fin loss, and craniofacial morphology in Osteichthyes
Source: BMC Evol Biol. 2010 Jan 25;10:21. doi: 10.1186/1471-2148-10-21 (PMC2825197; doi:10.1186/1471-2148-10-21)
Supplement: Additional file 3 — Landmark definition for morphometric analysis. (A) Polypterus palmas buettikoferi in dorsal view, (B) P. endlicheri congicus in dorsal view, (C) P. p. buettikoferi in lateral view, (D) P. e. congicus in lateral view. [file 1471-2148-10-21-S3.PDF]

Additional file 3

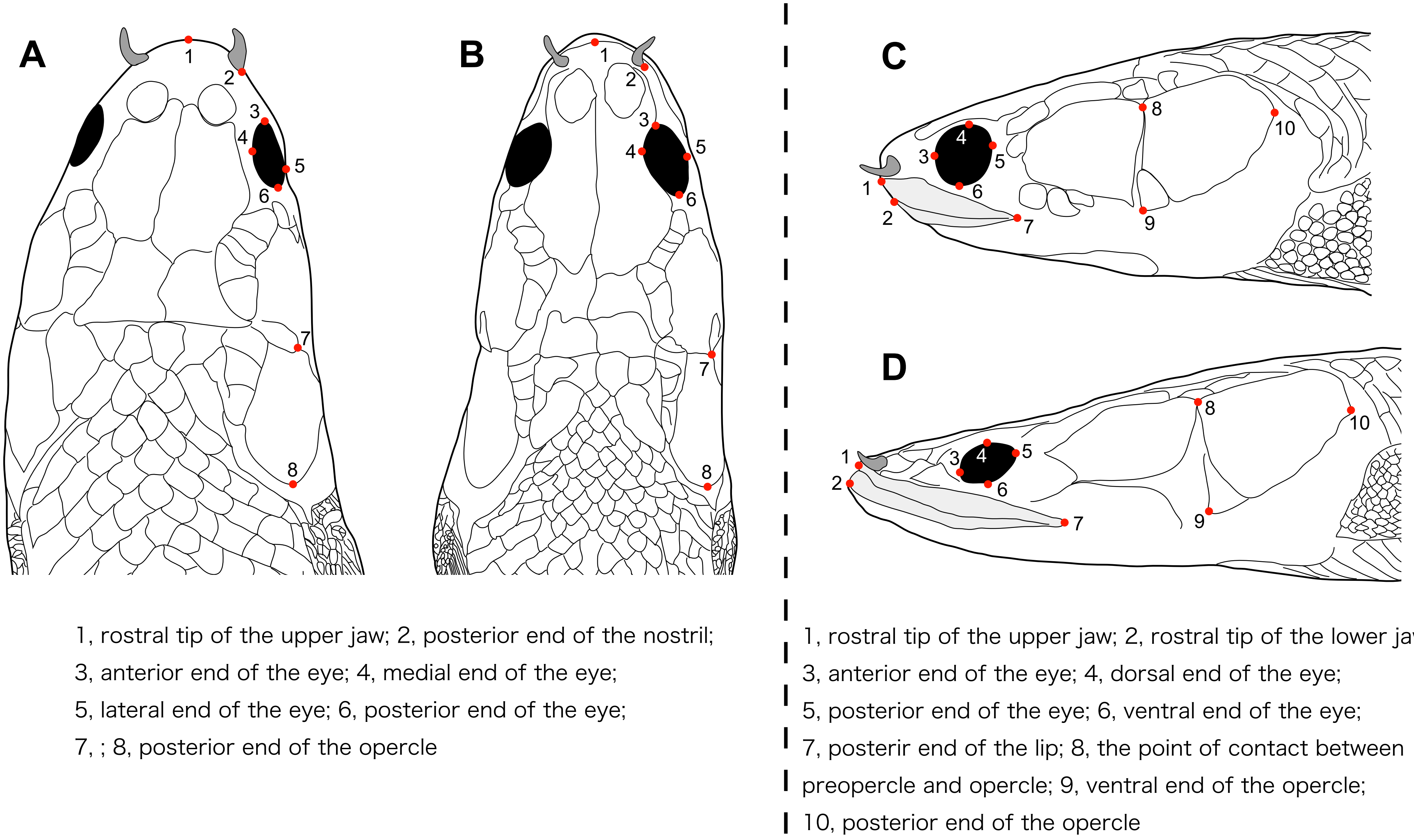

1, rostral tip of the upper jaw; 2, posterior end of the nostril;
3, anterior end of the eye; 4, medial end of the eye;
5, lateral end of the eye; 6, posterior end of the eye;
7, ; 8, posterior end of the opercle

1, rostral tip of the upper jaw; 2, rostral tip of the lower jaw;
3, anterior end of the eye; 4, dorsal end of the eye;
5, posterior end of the eye; 6, ventral end of the eye;
7, posterir end of the lip; 8, the point of contact between preopercle and opercle; 9, ventral end of the opercle;
10, posterior end of the opercle
