# Supplementary figures and images for: The mitochondrial phylogeny of an ancient lineage of ray-finned fishes (Polypteridae) with implications for the evolution of body elongation, pelvic fin loss, and craniofacial morphology in Osteichthyes
Source: BMC Evol Biol. 2010 Jan 25;10:21. doi: 10.1186/1471-2148-10-21 (PMC2825197; doi:10.1186/1471-2148-10-21)

**A**

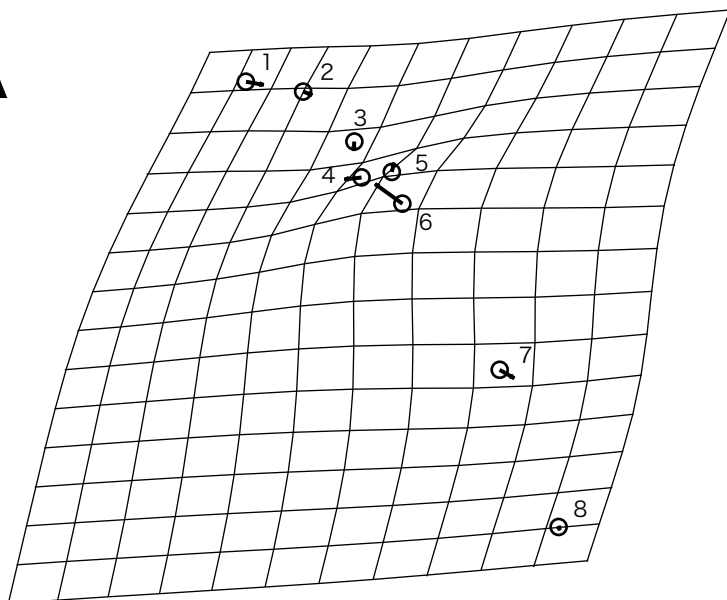

**C**

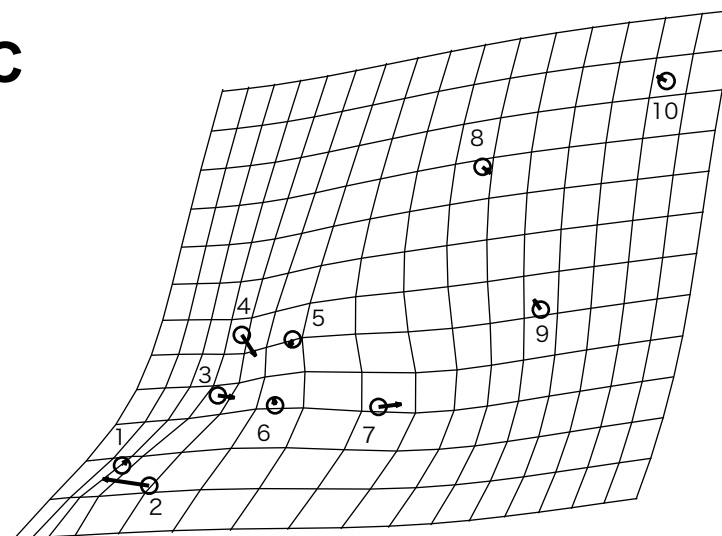

**B**

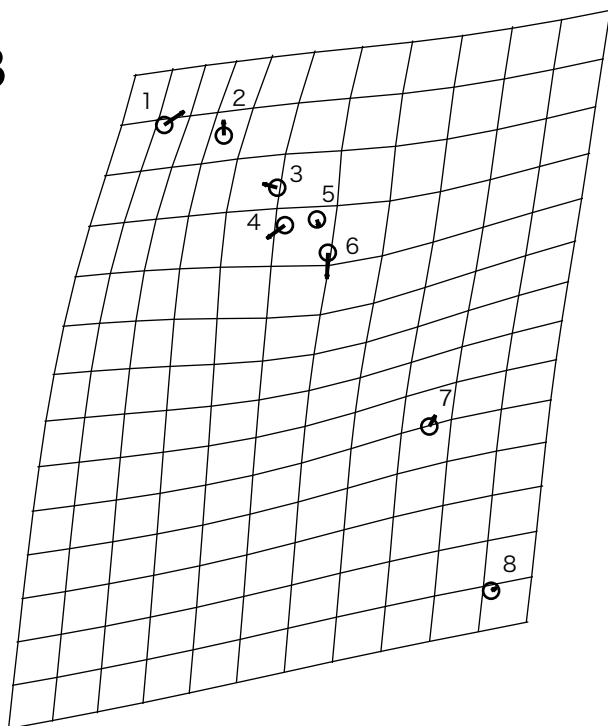

**D**

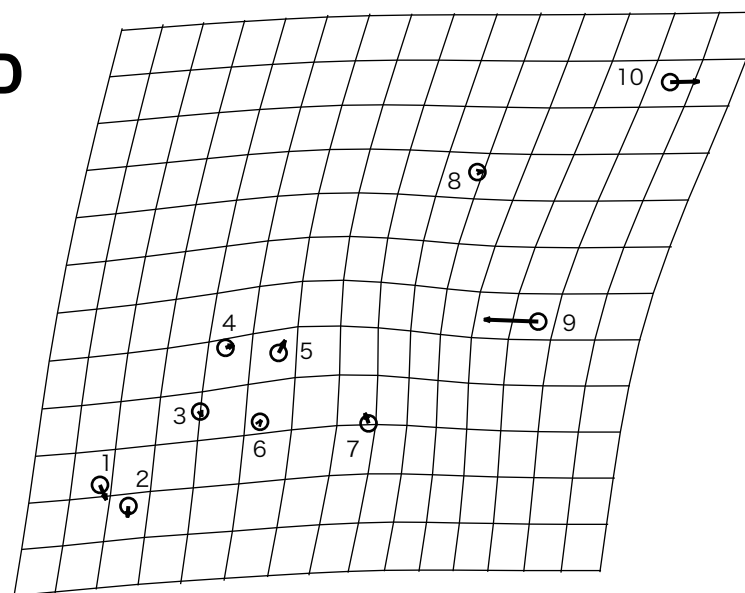

Supplement: Additional file 4 — Thin-plate splines (TPS) of principal component 1 (PC1) and 2 (PC2) for both dorsal and ventral views of the head of Polypterus. Arrows indicate PC value plus positive 0.1 score. (A) PC1 in dorsal view, (B) PC2 in dorsal view, (C) PC1 in lateral view, (D) PC2 in lateral view. Numbers along with each plot indicate landmarks defined in Additional file 3. [file 1471-2148-10-21-S4.PDF]
